# Social Media Effect on Personal Self-Esteem Among the Population in Saudi Arabia

**DOI:** 10.7759/cureus.49543

**Published:** 2023-11-28

**Authors:** Omar Ahmed M Alshaikhi, Saleh A Alshaikhi, Hassan Ali A AlZubaidi, Muslih Abdullah A Alzubaidi, Hassan Mohammed H Alfaqih, Ahmed Ali A Alrezqi, Mohsen Hashem S AlRashdi, Abdullah Ali A Alzubaidi, Mohannad Ahmed M Alshaikhi, Ramy M Ghazy, Ayoub A Alshaikh

**Affiliations:** 1 Department of Family Medicine, Ministry of Health, AlQunfudah, SAU; 2 Department of Family Medicine and Diabetes Management, Ministry of Health, AlQunfudah, SAU; 3 Department of Medicine and Surgery, Umm Al Qura University, AlQunfudah, SAU; 4 Department of Tropical Health, High Institute of Public Health, Alexandria University, Alexandria, EGY; 5 Department of Family & Community Medicine, King Khalid University, Abha, SAU

**Keywords:** social media platform, rosenberg self-esteem scale, social media use, saudi population, self-esteem

## Abstract

Background

Self-esteem is a self-valuation; it is how people perceive their own worth and how valuable they believe they are to others. In this study, our primary objective was to explore the association between social media use and self-esteem among individuals who actively engage with social media platforms in Saudi Arabia.

Method

This study involved individuals aged 15 and above who are active social media users residing in Saudi Arabia. The data were collected through an anonymous online cross-sectional survey. Participants were recruited using snowball and convenience sampling method. A questionnaire was administered through Google Forms to collect data from participants. The questionnaire was structured into three sections, which included gathering social and demographic information, assessing personal usage patterns, and evaluating individual self-esteem levels using an Arabic valid version of the Rosenberg Self-Esteem Scale.

Results

The survey included a total of 2,551 participants. Among them, 51.3% (n = 1,309) were female, 29% (n = 741) fell within the 21-25 age group, 95.7% (n = 2,441) were of Saudi nationality, and 51.6% (n = 1,316) were single. The social media platform most frequently used by participants was TikTok 98.5% (n = 2,512), followed by Facebook 95.7% (n = 2,441), Telegram 89.8% (n = 2,291), YouTube 72.2% (n = 1,942), WhatsApp 66.0% (n = 1,683), and finally, Snapchat 30.7% (n = 1,769). In total, 14.3% (n= 366) have low self-esteem, participants scored around 16.10 ± 1.80, ranging from 5 to 25. The following variables were significantly associated with self-esteem: female sex (83.88% vs 87.52%, *X*^2^ = 6.87, p = 0.009), nationality (*X*^2^ = 13.507, p < 0.001), marital status (*X*^2^ = 12.313, p = 0.006), region (*X*^2^ = 18.36, p = 0.001), using Tik Tok (*X*^2^ = 4.11, p = 0.043), the frequency of posting comments (*X*^2 ^= 12.01, p = 0.017), comparing oneself to others (*X*^2^ = 27.94, p < 0.001), using social media because of weak personal communication (*X*^2 ^= 6.56, p = 0.010), using social media to follow news (*X*^2 ^= 6.89, p = 0.009), and the perceived effect of social media (*X*^2 ^= 16.28, p < 0.001).

Conclusions

Our findings revealed that a minority of participants exhibited low self-esteem, and individuals from the Northern region were more likely to report such issues. Sociodemographic factors, including gender, nationality, and marital status, demonstrated associations with self-esteem. Additionally, the frequency of comments, TikTok usage, and peer comparison significantly influenced self-esteem levels.

## Introduction

One of the most popular online activities is using social media. The term "social media" refers to "a group of Internet-based applications that build on the ideological and technological foundations of the World Wide Web and enable the creation and exchange of user-generated content." Without a doubt, social media and user-generated content have become realities for millions of people and businesses [1]. Over 4.59 billion people used social media worldwide in 2022, with that figure expected to rise to nearly six billion by 2027 [2]. In fact, social media statistics show a 10% annual increase in the total number of handlers. Researchers are interested in investigating this marvel and its impact on every aspect of users' lives because of these massively imposing statistics [3].

The use of social media accounts such as Facebook, Snapchat, Instagram, and others has increased dramatically in recent years [4]. According to studies, 93-97% of adolescents aged 13-17 use at least one social media platform and spend approximately 3 hours per day on social media platforms [5-7]. Because social media users create, share, and/or exchange information and ideas in virtual communities, they can network with other members who have similar or shared interests, dreams, and goals [8].

One of the most commonly studied paradigms in youth is self-esteem. Self-esteem is frequently defined as an individual's negative or positive perception of their self-worth, sense of pride, positive self-evaluation, or self-respect [8]. It is also defined as the positive (high self-esteem) or negative (low self-esteem) feelings that we have about ourselves. We experience positive feelings of high self-esteem when we believe that we are good and worthy and that others view us positively. We experience negative feelings of low self-esteem when we believe that we are inadequate and less worthy than others. Self-esteem drives many changes throughout the lifespan, according to research, and it is high in childhood, decreases significantly during adolescence, increases in adulthood, becomes steadier in middle adulthood, and then decays again in old age [9].

According to MacIntyre et al. [10], self-esteem can be defined as an individual's self-evaluation. It represents how people perceive their self-worth and how much value they believe they hold in the eyes of others. Many studies have been conducted to investigate the relationship between self-esteem and communicative behaviors [10-12]. People with low self-esteem are less likely to engage in communication than those with high self-esteem because they believe they have less to contribute to the conversation and are more likely to receive negative feedback from others [10,12]. Furthermore, research shows that people with low self-esteem spend more time using instant messaging rather than face-to-face communication because they find communicating with others through technology easier than face-to-face communication [12]. It is worth noting that addictive social media use has been linked to low self-esteem [4,13]. Furthermore, research has shown that individuals who spend more time on social media tend to engage in upward social comparison, a behavior that can have adverse effects, especially among young people [14].

In this study, our primary objective was to explore the correlation between social media usage and self-esteem among individuals who actively engage with social media platforms in Saudi Arabia. In addition, we aimed to explore this relationship further by investigating variations across various regions within the Kingdom, as well as among individuals with different durations of social media usage.

## Materials and methods

Study design

This research adopts an anonymous online cross-sectional study design. The study was conducted between June 1, 2023, and July 15, 2023.

Target population

The study's focus on social media users aged 15 and older residing in Saudi Arabia aligns with a clear demographic target. Using Epi-info 7.4, the calculated minimum required sample size to detect a prevalence of 50% low self-esteem within the Saudi population who are using social media was determined to be 384. This calculation considered a precision of 5% and a confidence interval of 95%. The sample size was increased by a factor of 5 to address the effects of stratification, and it was further increased by 30% to compensate for non-response. The study participants were recruited using the snowball and convenience sampling method.

Study setting

Data collection was carried out within Saudi Arabia. Saudi Arabia consists of five regions: the Central region, the Eastern region, the Northern region, the Southern region, and the Western region. All these regions were presented in this survey. 

Data collection

To collect data, we uploaded the questionnaire to Google form and the link was distributed through various social media platforms such as WhatsApp, Snapchat, and Twitter, among others. Before data collection, the questionnaire underwent a pilot phase. Each author was tasked with collecting feedback from five individuals to assess the time required to complete the questionnaire and the clarity of the wording. The responses obtained during the pilot phase were not included in the final analysis.

The questionnaire used in this study consists of three sections. The first section of the questionnaire focuses on collecting social and demographic information, including age (grouped into categories of 15-20 years, 21-25 years, 26-30 years, 31-35 years, 36-40 years, and above 40 years), gender (male or female), nationality (Saudi and non-Saudi), marital status (single, married, divorced, widowed), region (Central, Eastern, Northern, Southern, and Western), income level (below 5000 SAR, between 5000 and 10,000 SAR, and above 10,000 SAR), and educational level (ranging from illiterate, primary, preparatory, secondary, university degree, to postgraduate degrees including master's and doctorate degrees). 

The second section assesses the personal usage patterns of social media, including type of social media used (YouTube, Telegram, Facebook, WhatsApp, Snapshot, and TikTok), frequency of utilization (half an hour or less, 1-3 hours daily, 3-5 hours daily, more than 5 hours daily), frequency of posting comments (daily, weekly, monthly, yearly, never), and causes of social media use (making friends, work and trade, education and learning, free time, time loss, entertainments, due to problems with face-to-face communication, build relationship, communication, and following news). 

The third section evaluates individual self-esteem levels. A 10-item scale that assesses overall self-esteem by assessing both positive and negative feelings about oneself. The scale is thought to be one-dimensional. All items are graded on a 4-point Likert scale from strongly agree to strongly disagree [15].

Ethical consideration

All participants received detailed information on the objectives and purpose of the study, ensuring that they had the autonomy to make an informed decision about whether or not to provide their consent and participate in the study. The research team placed a paramount emphasis on protecting the confidentiality and anonymity of participant data throughout the research process. In accordance with the principles outlined in the Declaration of Helsinki, ethical approval was obtained from the University of King Khalid Research Ethics Committee with approval number: ECM#2023-1602ECM#2023-1602.

Statistical analysis

Rosenberg Self-Esteem Scale Interpretation

The scoring system for this assessment ranges from a minimum total score of 0 to a maximum score of 30, where higher scores indicate higher levels of self-esteem. The results include four raw scores and four percentiles, each offering unique insights into the participant's self-esteem. Scores between 15 and 25 are considered normal; scores below 15 indicate low self-esteem. Self-competence: It is calculated by summing the scores of the first five items in the questionnaire, providing an indication of one's perception of their competence. Self-liking: This score is derived from the sum of the second five items, shedding light on how much an individual likes themselves. Self-competence minus self-liking (SC-SL): This score represents the difference between self-competence and self-liking scores, offering a nuanced perspective on the balance between self-confidence and self-regard. The data was meticulously entered into Excel 2016 and subjected to statistical analysis using Statistical Package for Social Sciences (SPSS) software, version 26 (IBM Corp, Armonk, NY). We presented numerical data using means and standard deviations, while categorical variables were described using numbers and percentages. To assess the association between categorical variables, we used the chi-square test. A significance level of P <0.05 was used to determine statistical significance in the analysis.

## Results

Table 1 provides describes several key demographic variables of the studied population. A total of 2,551 participants were included in this survey. There was a fairly balanced gender distribution, with 51.3% (n = 1,309) female and 48.7% (n = 1,242) male respondents. The age distribution of the respondents is quite diverse. The largest age group falls in the 21-25 years category 29.0% (n = 741), followed by above 40 years 22.4% (n = 572). The vast majority of respondents were Saudi nationals 95.7% (n = 2,441), while a small percentage 4.3% (n = 110) were non-Saudi. The majority of respondents were either single 51.6% (n = 1,316) or married 44.5% (n = 1,135), with smaller percentages being divorced 2.4% (n = 60) or widowed 1.6% (n = 40). The table shows the distribution of respondents across different regions. The Central region has the highest representation 29.9% (n = 762), followed closely by the Eastern region 27.4% (n = 699). A significant proportion of respondents had a bachelor's degree 62.8% (n = 1,601), followed by secondary education 22.6% (n = 576). The income distribution was divided into three categories, with the highest percentage falling in the "Less than 5000 SAR" category at 43.8% (n = 1,118).

**Table 1:** Demographic profile of surveyed population Numerical data are presented as mean ± SD; categorical data are presented as number (%).

Studied variables (n = 2,551)		Frequency	Percent
Gender	Female	1309	51.3
	Male	1242	48.7
Age	15-20 years	331	13.0
	21-25 years	741	29.0
	26-30 years	418	16.4
	31-35 years	239	9.4
	36-40 years	250	9.8
	Above 40 years	572	22.4
Nationality	Non-Saudi	110	4.3
	Saudi	2441	95.7
Marital status	Divorced	60	2.4
	Single	1316	51.6
	Widow	40	1.6
	Married	1135	44.5
Region of residence	Central region	762	29.9
	Eastern region	699	27.4
	Northern region	298	11.7
	Southern region	518	20.3
	Western region	274	10.7
Educational level	Illiterate	27	1.1
	Primary education	35	1.4
	Preparatory education	56	2.2
	Secondary education	576	22.6
	Bachelor's degree	1601	62.8
	Master's degree	197	7.7
	Doctorate degree	59	2.3
Income level	<5000 SAR	1118	43.8
	1000-5000 SAR	656	25.7
	More than 1000 SAR	777	30.5

**Figure 1:**
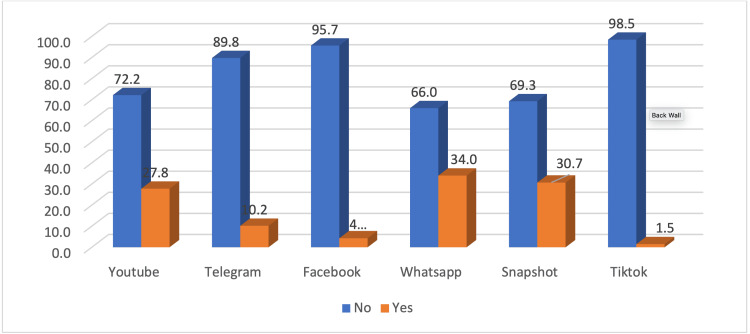
The distribution of different social media utilization among the surveyed population Categorical data are presented as number (%).

The most commonly used social media platform was TikTok 98.5% (n = 2,512), followed by Facebook 95.7% (n = 2,441), Telegram 89.8% (n = 2,291), YouTube 72.2% (n = 1,942), WhatsApp 66.0% (n = 1,683), and finally Snapshot 30.7% (n = 1,769) (Figure 1).

Table 2 provides an overview of several variables related to social media usage among the studied group of individuals. The majority of respondents spent a significant amount of time on social media, with 30.6% (n = 781) using it for 1 to 3 hours daily and 33.6% (n = 856) spending 3 to 5 hours daily. Approximately, one-third 30.1% (n = 769) spent more than 5 hours per day on social media. On the other hand, 5.7% (n = 145) of respondents spent only half an hour or less on social media. The frequency of posting comments varies, with the majority doing so either daily 24.3% (n = 620) or weekly 26.3% (n = 670). A large number of respondents 29.2% (n = 746) never post comments on social media. The reasons for using social media were diverse. The most prominent reasons seem to be for communication 35.8% (n = 913) and staying updated on news 50.9% (n = 1,298). It is noteworthy that a small percentage of respondents used social media to make friends, for work or trade, or educational purposes, suggesting a variety of motivations for social media usage. A small sector of the participants 3.1% (n = 98) used social media because of problems with face-to-face communication.

**Table 2:** Frequency and causes of social media utilization among the surveyed sample (n = 2,551) Numerical data are presented as mean ± SD; categorical data are presented as number (%).

Variables		No	%
Time spent on social media	Half an hour or less	145	5.7
	1-3 hours/day	781	30.6
	3-5 hours/day	856	33.6
	More than 5 hours/day	769	30.1
Posting comments	Daily	620	24.3
	Weekly	670	26.3
	Monthly	368	14.4
	Yearly	147	5.8
	Never	746	29.2
Causes of social media utilization	Make friends	2	0.1
	Work or trade	4	0.2
	Education and learning	12	0.5
	Free time	37	1.4
	Entertainment	29	1.1
	Due to problems with face-to-face communication	78	3.1
	Build relationship	114	4.5
	Communication	913	35.8
	News	1298	50.9

**Figure 2:**
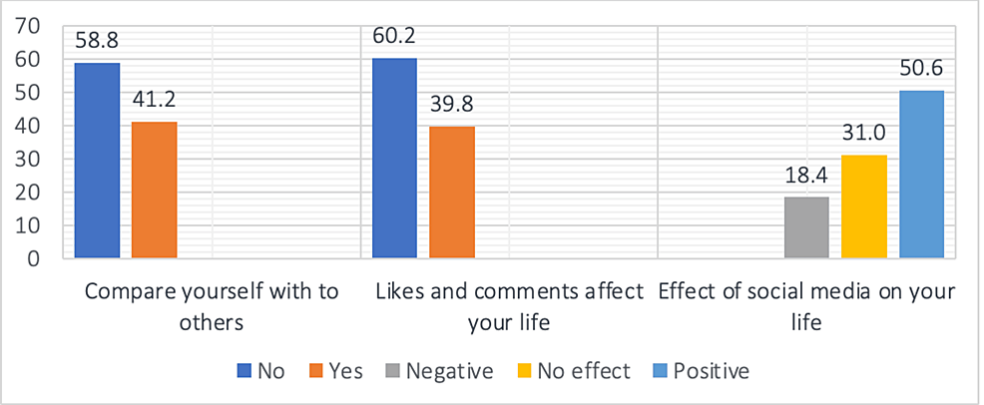
Participant self-perception Categorical data are presented as number (%).

Figure 2 shows that 41.2% (n = 1052) compare themselves with others, and 39.8% (n = 1015) confessed that liking their posts significantly affected their life. Generally, 50.6% (n = 1,290) of the participants reported that social media positively impacted their life, while 18.4% (n = 470) thought it negatively affected their life.

Table 3 presents a summary of the statistical values for the self-esteem scores among the participants. The minimum total score achieved by participants was 5.00, while the maximum was 25.00. On average, participants scored around 16.10, with a standard deviation of 1.80. The self-competence scores ranged from a minimum of 3.00 to a maximum of 14.00. The mean score for self-competence was 8.80, with a standard deviation of 1.14. Participants' self-liking scores varied from a minimum of 2.00 to a maximum of 15.00. The mean score for self-liking was 7.30, with a standard deviation of 1.29. The range for self-competence minus self-liking scores was from -7.00 to 8.00, with a mean score of 1.50 and a standard deviation of 1.65. 

**Table 3:** The self-esteem questionnaire scores among the surveyed population Numerical data are presented as mean ± SD.

Self-esteem scores	Minimum	Maximum	Mean	Standard deviation
Total score	5.00	25.00	16.10	1.80
Self-competence	3.00	14.00	8.80	1.14
Self-liking	2.00	15.00	7.30	1.29
Self-competence minus self-liking	-7.00	8.00	1.50	1.65

Table 4 shows that 14.3% have low self-esteem. There was a statistically significant association between gender and self-esteem (χ^2^ = 6.869, p = 0.009). Females were more likely to have low self-esteem compared to males (16.12% vs 12.48%, χ^2^ = 6.87, p = 0.009). Although there were differences in self-esteem in age categories, the chi-square test did not show a statistically significant association (p = 0.056). However, specific age groups, such as those aged 21-25 years, exhibit the highest magnitude of low self-esteem compared to others. There was a highly significant association between nationality and self-esteem (χ^2^ = 13.507, p < 0.001). Non-Saudi nationals were more likely to report low self-esteem compared to Saudi nationals (26.36% vs 13.81%). Marital status was significantly associated with self-esteem (χ^2^ = 12.313, p = 0.006). Widowed individuals were more likely to have low self-esteem compared to other marital status categories (22.50%). There was a significant association between region and self-esteem (χ^2^ = 18.357, p = 0.001). The table indicates that individuals from the Northern region were more likely to report low self-esteem issues compared to other regions. Although there was no statistically significant overall association between income level and self-esteem (p = 0.052), the table shows that those with monthly income below 5000 SAR were more likely to have low self-esteem.

**Table 4:** Variables affecting self-esteem χ^2^: chi-square; numerical data are presented as mean ± SD; categorical data are presented as number (%).

Studied variables	Level	Low self-esteem				X^2^	p-Value
		Yes (n = 366)	%	No (n = 2185)			
		n	%	n		%	
Gender	Female	211	16.12	1098	83.88		
	Male	155	12.48	1087	87.52	6.87	0.009
Age	15-20 years	53	16.01	278	83.99		
	21-25 years	120	16.19	621	83.81		
	26-30 years	64	15.31	354	84.69	10.784	0.056
	31-35 years	37	15.48	202	84.52		
	36-40 years	32	12.80	218	87.20		
	Above 40 years	60	10.49	512	89.51		
Nationality	Non-Saudi	29	26.36	81	73.64	13.507	0.0001
	Saudi	337	13.81	2104	86.19		
Marital status	Divorced	7	11.67	53	88.33	12.313	0.006
	Married	135	11.89	1000	88.11		
	Single	215	16.34	1101	83.66		
	Widow	9	22.50	31	77.50		
Region	Central region	109	14.30	653	85.70	18.357	0.001
	Eastern region	87	12.45	612	87.55		
	Northern region	65	21.81	233	78.19		
	Southern region	62	11.97	456	88.03		
	Western region	43	15.69	231	84.31		
Income level	<5000 SAR	176	15.74	942	84.26	5.931	0.052
	1000-5000 SAR	98	14.94	558	85.06		
	More than 1000 SAR	92	11.84	685	88.16		

Table 5 presents a cross-tabulation of various studied variables, including self-esteem, TikTok usage, posting comments frequency, comparison with others, experiencing weak communication, using social media for news, and the perceived effect of social media. About 25.64% of those who were using TikTok had low self-esteem compared to 14.17% of those who were not using TikTok (χ^2 ^= 4.11, p = 0.043). There was a significant association between self-esteem and the frequency of posting comments (χ^2 ^= 12.01, p = 0.017). Those who were posting comments daily had the highest prevalence of low self-esteem (17.74%). There was a strong association between self-esteem and comparing oneself to others. Individuals who compared themselves to others had a higher prevalence of low self-esteem compared to their peers (18.73% vs 11.27%) (27.94, p < 0.0001). A significant association exists between self-esteem and using social media as they experienced weak personal communication (χ^2^ = 6.56, p = 0.010). There was a significant association between self-esteem and using social media to follow the news (χ^2 ^= 6.89, p = 0.009). Individuals who were using social media for following news had a lower prevalence of self-esteem compared to their peers (12.56% vs. 16.20%). There was a highly significant association between self-esteem and the perceived effect of social media (χ^2^ = 16.28, p < 0.001). Those who perceived the positive impact of social media had a low prevalence of self-esteem compared to those who did not (13.26% vs 20.21%).

**Table 5:** Association between self-esteem and social media behavior χ^2^: chi-square; numerical data are presented as mean ± SD; categorical data are presented as number (%).

Studied variables		Self-esteem				Test statistics	X^2^
		No (n = 366)		Yes (n = 2185)			
TikTok	No	356	14.17	2156	85.83	4.11	0.043
	Yes	10	25.64	29	74.36		
Posting comments	Daily	110	17.74	510	82.26	12.01	0.017
	Weekly	102	15.22	568	84.78		
	Monthly	50	13.59	318	86.41		
	Yearly	15	10.20	132	89.80		
	Never	89	11.93	657	88.07		
Time spent on social media	Half an hour or less	18	12.41	127	87.59	5.11	0.164
	1-3 hours/day	98	12.55	683	87.45		
	3-5 hours/day	124	14.49	732	85.51		
	More than 5 hours/day	126	16.38	643	83.62		
Compare yourself to others	No	169	11.27	1330	88.73	27.94	0.0001
	Yes	197	18.73	855	81.27		
Weak communication	No	19	24.36	59	75.64	6.56	0.010
	Yes	366	14.35	2185	85.65		
Use social media for news	No	203	16.20	1050	83.80	6.89	0.009
	Yes	163	12.56	1135	87.44		
Effect of social media	Negative	95	20.21	375	79.79	16.28	0.0001
	No effect	100	12.64	691	87.36		
	Positive	171	13.26	1119	86.74		

## Discussion

In this study, our objective was to evaluate the levels of self-esteem among social media users in Saudi Arabia. To achieve this, we gathered our sample from all five regions of Saudi Arabia using a combination of convenience and snowballing sampling methods. We found that the mean total score was 16.10 ± 1.80. The mean score for self-competence was 8.80 ± 1.14. The mean score for self-liking was 7.30 ± 1.29. Among the study participants, 14.3% had low self-esteem. The mean score of self-competence minus self-liking was 1.50 ± 1.65. Individuals from the Northern region were more likely to report low self-esteem compared to those from other regions. There was no significant difference regarding the time spent using social media and self-esteem. The main sociodemographic factors associated with self-esteem were gender, nationality, and marital status, while the frequency of posting comments, time spent on social media, using TikTok, and comparing subjects with their peers significantly affected self-esteem. 

Causes of utilization of social media

While social media is frequently lauded as a cure for loneliness, a growing body of evidence suggests that it may actually worsen feelings of isolation. Unsurprisingly, it can set off a dangerous game of comparison, leaving people doubting their self-worth [16]. The use of social media is strongly linked to the development of anxiety and other psychological problems such as depression, insomnia, stress, lower subjective happiness, and a sense of mental deprivation [17]. In this study, we found that the most common cause of social media use was following news and communication. We found that nearly one-third of the participants spent more than 5 hours per day using social media, and 33.6% were spending between 3 and 5 hours per day. This finding indicates that a large number are using social media for a long duration. However, we did not find any significant association between the duration of social media use and self-esteem. The social media addiction should be assessed in future studies in Saudi Arabia. Social media addiction can be characterized as a form of behavioral addiction, involving compulsive and excessive involvement in social media platforms. This addiction can have a substantial impact on the users' functioning in critical life areas, including interpersonal relationships, work or academic performance, and physical health [18].

The self-esteem 

The mean self-esteem score of the studied participants was 16.1 ± 1.8 with 14.3% having low self-esteem (scored below 15). This finding suggests that Saudi patients using social media experienced minimal effect on self-esteem. Another evidence was provided by a study among 387 adolescents which found that 88% experienced no or very small effects of social media use on self-esteem, while 4% experienced positive and 8% negative effects [19]. On the other hand, Woods and Scott [20] discovered that adolescents with high levels of social media use had low self-esteem in a study with 467 adolescents. Moreover, Jan et al. discovered a negative relationship between their participants' daily social media usage times and their self-esteem levels [19]. Robust evidence was provided by a meta-analysis study conducted by Liu and Baumeister. They looked at 33 studies published between 2008 and 2016. In the study, it was reported that there was a negative relationship between social media use and self-esteem [21]. Indeed, social media addiction negatively predicted self-esteem and body image levels as well. Many studies assessed the effect of social media on self-perception of body image. Colak et al. [13] examined the relationship between self-esteem, social media addiction, and body image in high school students. There was a negative moderate relationship between self-esteem and social media addiction levels and a positive correlation between self-esteem and body image. Similarly, a study involving 150 students from the Institute of Business Management shed light on the impact of upward comparisons made on social networking sites, with a particular focus on Facebook. The research highlighted that these upward comparisons on Facebook can lead to reduced self-esteem, with a significant portion of users, 88%, engaging in such comparisons, and the majority, 98%, involving upward comparisons [22]. 

Association between age and sex with self-esteem

Age

We found that older age groups among social media users had higher self-esteem; however, this difference was not statistically significant (p = 0.056). Likewise, Kavas [23] reported that self-esteem among social media users was not affected by age. On the other hand, Steinsbekk et al. [24] found that increased social media use predicted decreased appearance self-esteem from age 10-12 years and from age 12-14 years.

Gender

Regarding sex, we found that male sex was also associated with higher self-esteem. A similar finding was reported by Steinsbekk et al. [24]. They found a significant gender-based difference: the impact of other-oriented social media use on appearance self-esteem was significant in girls but absent in boys [13].

Level of Education

We found that the level of education was not significantly associated with self-esteem among social media users. Our finding was consistent with the finding of Colak et al. [13]. On the other hand, Özcan et al. [25] showed that there was a significant relationship between education levels and the self-esteem levels of adolescents, and as the education levels increase, self-esteem levels also increase.

Nationality and Residence 

We found that the Saudi population using social media had higher self-esteem compared to their peers who did not belong to Saudi. Regarding Saudi regions, we found that those who were living in the northern region significantly had a higher prevalence of low self-esteem compared to those who were living in other Saudi regions. 

Strengths and limitations

To our knowledge, this is the first study to be conducted in Saudi Arabia to address the impact of social media on self-esteem. However, limitations include the potential for bias due to snowball and convenience sampling, reliance on self-reported data susceptible to response bias, a cross-sectional design unable to establish causation, and the use of a one-dimensional Likert scale for assessing self-esteem. Additionally, the study may not fully address cultural differences among non-Saudi participants and lacks extensive contextual information, which should be considered when interpreting its findings. Furthermore, it is crucial to acknowledge that various confounding factors may potentially come into play and influence the relationship between the dependent variable, self-esteem, and the variables under investigation in our study. Finally, it is important to note that we did not specifically investigate mental health issues, such as depression, anxiety, and stress, as potential factors driving social media usage in our study.

## Conclusions

In conclusion, this study offers valuable insights into the relationship between social media use and self-esteem among a specific demographic of users in Saudi Arabia. The study's findings revealed that a small sector of the study participants exhibited low self-esteem. Interestingly, individuals from the Southern region exhibited higher self-esteem issues when compared to those from other regions. Moreover, the study did not find a significant association between the time spent using social media and self-esteem. However, key sociodemographic factors like gender, nationality, and marital status were associated with self-esteem, while factors such as the frequency of posting comments, the use of TikTok, and comparing oneself with peers had a significant impact on self-esteem. These findings highlight the complex interplay of various factors influencing self-esteem in social media users in Saudi Arabia and emphasize the importance of considering regional, sociodemographic, and online behavioral aspects in further research and interventions related to self-esteem in this context.
